# Mitochondrial dysfunction in sepsis-induced immunoparalysis: from immune-cell metabolic reprogramming to clinical biomarkers

**DOI:** 10.3389/fimmu.2026.1904513

**Published:** 2026-07-31

**Authors:** Jiaxin Sun, Yuye Yang, Shiyun Long, Xinsen Zou, Chenyang Duan, Yanqing Wang

**Affiliations:** 1 Department of Anesthesiology, Sichuan Clinical Research Center for Cancer, Sichuan Cancer Hospital & Institute, Sichuan Cancer Center, University of Electronic Science and Technology of China, Chengdu, China; 2 Department of Critical Care Medicine, The Second Affiliated Hospital of Chongqing Medical University, Chongqing, China; 3 Department of Critical Care Medicine, Bishan Hospital of Chongqing Medical University, Chongqing, China

**Keywords:** biomarkers, immune metabolism, immunoparalysis, mitochondrial dysfunction, sepsis

## Abstract

Sepsis-induced immunoparalysis is a dynamic state of acquired immune dysfunction characterized by impaired antigen presentation, lymphocyte exhaustion, defective innate immune responses, and increased susceptibility to secondary infection. Increasing evidence suggests that mitochondrial dysfunction is a key metabolic mechanism underlying this immune failure. Rather than acting as a uniform injury signal, mitochondrial abnormalities affect immune-cell subsets in distinct ways: monocytes and macrophages lose antigen-presenting capacity, neutrophils develop impaired migration and antimicrobial activity, T cells acquire exhaustion-like phenotypes, and NK and B-cell responses become functionally constrained. This review summarizes how core mitochondrial processes, including bioenergetic failure, redox imbalance, mitochondrial danger-signal release, and defective quality control, contribute to sepsis-induced immunoparalysis. We further discuss how mitochondria-related readouts may complement established immune markers such as monocyte HLA-DR, lymphocyte count, PD-1/PD-L1, CD86, and IL-10 for patient stratification. Finally, we highlight therapeutic opportunities aimed at restoring mitochondrial fitness and immune competence in biomarker-defined septic patients.

## Highlights

- Mitochondrial dysfunction links immune-cell metabolic failure to sepsis-induced immunoparalysis.
- Mitochondrial and immune biomarkers may improve early identification of immunosuppressed septic patients.
- Targeting mitophagy, mitochondrial ROS, and immune checkpoints may restore host immune competence.

## 1 Introduction

Sepsis is a life-threatening syndrome caused by a dysregulated host response to infection and remains a major cause of mortality in intensive care units worldwide (1–5). Although early sepsis is traditionally characterized by excessive systemic inflammation, it is now increasingly recognized that the immune response in sepsis is highly dynamic and heterogeneous (6–8). Many patients do not follow a simple linear course from hyperinflammation to recovery. Instead, pro-inflammatory activation and anti-inflammatory suppression may coexist, fluctuate over time, and vary substantially among individuals (9–13). This immunological complexity partly explains why broadly anti-inflammatory or immune-stimulatory therapies have produced inconsistent clinical benefits in unselected septic populations.

A key feature of this immune heterogeneity is sepsis-induced immunoparalysis, a state of acquired immune dysfunction that predisposes patients to persistent infection, secondary nosocomial infections, viral reactivation, impaired pathogen clearance, and prolonged organ failure (14–17). Immunoparalysis is commonly reflected by reduced monocyte human leukocyte antigen-DR expression, impaired antigen presentation, diminished ex vivo cytokine production after immune stimulation, lymphopenia, T-cell exhaustion, expansion of immunosuppressive cell populations, and dysfunction of natural killer cells and other innate immune effectors (18–21). The PROVIDE randomized clinical trial provided important clinical support for the concept that septic patients can be stratified according to immune phenotypes, including macrophage-activation-like syndrome and immunoparalysis, using biomarkers such as ferritin and monocyte HLA-DR expression. This trial also emphasized that immune-directed therapy is more likely to be effective when guided by host-response phenotyping rather than applied uniformly to all patients.

The recognition of sepsis endotypes has shifted the field from a “one-size-fits-all” model toward mechanism-based and biomarker-guided immunotherapy. Gómez and colleagues further highlighted that inflammatory phenotypes may help identify patients who are more likely to benefit from mechanism-specific interventions (22). In this context, immunoparalysis should not be viewed merely as passive immune exhaustion after an inflammatory storm. Rather, it represents an active and regulated pathological state involving profound changes in cellular metabolism, mitochondrial activity, redox balance, antigen-processing capacity, and immune checkpoint signaling. Recent evidence showing that PD-L1-positive plasma cells can suppress T-lymphocyte responses in patients with sepsis and experimental sepsis models further supports the idea that adaptive immune suppression is sustained by defined cellular and molecular circuits (23–27).

Mitochondria are increasingly recognized as central regulators of immune-cell fate and function (28). Beyond their classical role in ATP production, mitochondria control reactive oxygen species generation, calcium signaling, apoptosis, innate immune activation, inflammasome signaling, mitochondrial DNA release, and metabolic transitions between glycolysis, oxidative phosphorylation, and fatty acid oxidation. These processes are essential for immune-cell activation, differentiation, effector function, and resolution of inflammation (29, 30). During sepsis, mitochondrial injury can disrupt these programs, leading to impaired energy production, excessive oxidative stress, defective mitophagy, altered mitochondrial dynamics, and release of mitochondrial damage-associated molecular patterns (31–35). These alterations may contribute to or reflect monocyte deactivation, macrophage dysfunction, neutrophil dysregulation, T-cell exhaustion, lymphocyte apoptosis, and impaired natural killer cell activity, depending on the cell type and level of evidence.

Therefore, mitochondrial dysfunction provides a biologically plausible bridge between immune-cell metabolic reprogramming and sepsis-induced immunoparalysis. In monocytes and macrophages, impaired mitochondrial quality control may reduce antigen presentation and promote maladaptive inflammatory or tolerogenic states. In T cells, mitochondrial damage and metabolic insufficiency may limit clonal expansion, cytokine production, and cytotoxic function while promoting exhaustion-associated phenotypes (36–38). In neutrophils, mitochondrial-derived signals may influence chemotaxis, oxidative responses, cell death pathways, and interactions with endothelial or parenchymal cells. These cell-specific effects suggest that mitochondrial dysfunction is not only a consequence of severe infection but also an active driver of immune failure.

Importantly, mitochondrial alterations may also offer clinically measurable biomarkers for immune monitoring in sepsis. Circulating mitochondrial DNA, mitochondrial reactive oxygen species, mitochondrial membrane potential, oxygen consumption rate, peripheral immune-cell mitochondrial mass, and mitochondria-related gene signatures have all been explored as potential indicators of disease severity, immune dysfunction, or prognosis. When integrated with established immune markers such as monocyte HLA-DR, PD-1/PD-L1 expression, lymphocyte counts, cytokine profiles, and ex vivo immune-stimulation assays, mitochondrial biomarkers may help define a more precise immunometabolic profile of septic patients (39–41). Such a framework could support early identification of immunoparalysis, improve patient stratification, and guide individualized immunomodulatory or mitochondria-targeted interventions.

In this review, we summarize current evidence linking mitochondrial dysfunction to sepsis-induced immunoparalysis, with a particular focus on immune-cell metabolic reprogramming and clinically relevant biomarkers. To reduce descriptive repetition and improve conceptual progression, we first define the clinical and immunological features of immunoparalysis, then discuss shared mitochondrial mechanisms that disturb immune-cell function, and finally examine how these mechanisms produce cell-specific defects in innate and adaptive immunity. We further highlight emerging mitochondrial and immunometabolic biomarkers, discuss their translational potential, and outline therapeutic opportunities for precision sepsis care.

This narrative review summarizes current evidence linking mitochondrial dysfunction to sepsis-induced immunoparalysis. Relevant studies were identified from PubMed, Web of Science, Scopus, and Google Scholar using combinations of terms including “sepsis,” “septic shock,” “immunoparalysis,” “immunosuppression,” “mitochondrial dysfunction,” “immunometabolism,” “mitophagy,” “mtROS,” “mtDNA,” “PINK1/Parkin,” “NLRP3,” “HIF-1α,” “AMPK,” “mTOR,” “monocyte HLA-DR,” “PD-1/PD-L1,” and “biomarkers.” Human clinical studies and biomarker studies were prioritized for sections addressing diagnosis, prognosis, and patient stratification. Animal and *in vitro* studies were included to explain mechanisms such as mitochondrial damage, metabolic reprogramming, inflammasome activation, and immune-cell dysfunction. Recent publications were preferentially considered, while older landmark studies were cited when they established foundational concepts. Emerging therapeutic strategies were interpreted cautiously according to their level of evidence.

## 2 Sepsis-induced immunoparalysis: beyond hyperinflammation

### 2.1 Innate immune cell dysfunction

In sepsis, innate immune cells are among the first responders to invading pathogens, and their functional integrity is crucial for effective pathogen clearance (42, 43). However, during the transition from the initial hyperinflammatory phase to immunoparalysis, innate immune cells such as monocytes, macrophages, and neutrophils undergo profound functional alterations. Monocytes and macrophages frequently display reduced surface expression of human leukocyte antigen-DR (HLA-DR), which serves as a widely used clinical marker of impaired antigen presentation and immune competence (44–48). This reduction in HLA-DR is associated with diminished ex vivo cytokine production following stimulation, particularly of pro-inflammatory cytokines such as TNF-α and IL-12, while anti-inflammatory mediators such as IL-10 are elevated, reflecting a skewing toward a tolerogenic or immunosuppressive state (22, 49). These changes compromise the capacity of monocytes and macrophages to orchestrate effective adaptive immune responses, creating a permissive environment for secondary infections.

Neutrophils, another critical component of innate immunity, also exhibit dysfunction during immunoparalysis (50–54). Their chemotactic capacity is often impaired, limiting effective migration toward infection sites. In addition, aberrant formation of neutrophil extracellular traps (NETs) has been observed, which can paradoxically contribute to tissue damage while failing to enhance microbial clearance (55–59). Kwon and colleagues (2024) demonstrated that accumulation of circulating mitochondrial N-formyl peptides impairs neutrophil migration and reactive oxygen species (ROS) production, highlighting a mechanistic link between mitochondrial-derived danger signals and neutrophil dysfunction (60). Additional studies in both clinical and experimental sepsis models confirm that neutrophil energy depletion and oxidative stress further exacerbate these functional defects (61–63). Collectively, these alterations in innate immune cells are central to the establishment and maintenance of sepsis-induced immunoparalysis, as they reduce pathogen clearance and increase susceptibility to secondary infections and multi-organ dysfunction.

### 2.2 Adaptive immune cell dysfunction

Adaptive immune cells are similarly affected during immunoparalysis, with profound consequences for long-term immune competence. CD4+ and CD8+ T lymphocytes exhibit both quantitative and qualitative defects. Lymphopenia is a common feature, resulting from apoptosis and impaired proliferation, while surviving T cells often display metabolic insufficiency and exhaustion, characterized by reduced glycolytic capacity, diminished oxidative phosphorylation, and impaired effector cytokine production (64–67). Moreover, the expression of inhibitory receptors such as PD-1 is frequently upregulated, which further suppresses T-cell activation and cytotoxicity (68, 69). These changes not only compromise direct pathogen clearance but also impair the ability to provide help to B cells and coordinate the broader immune response.

**Figure 1:**
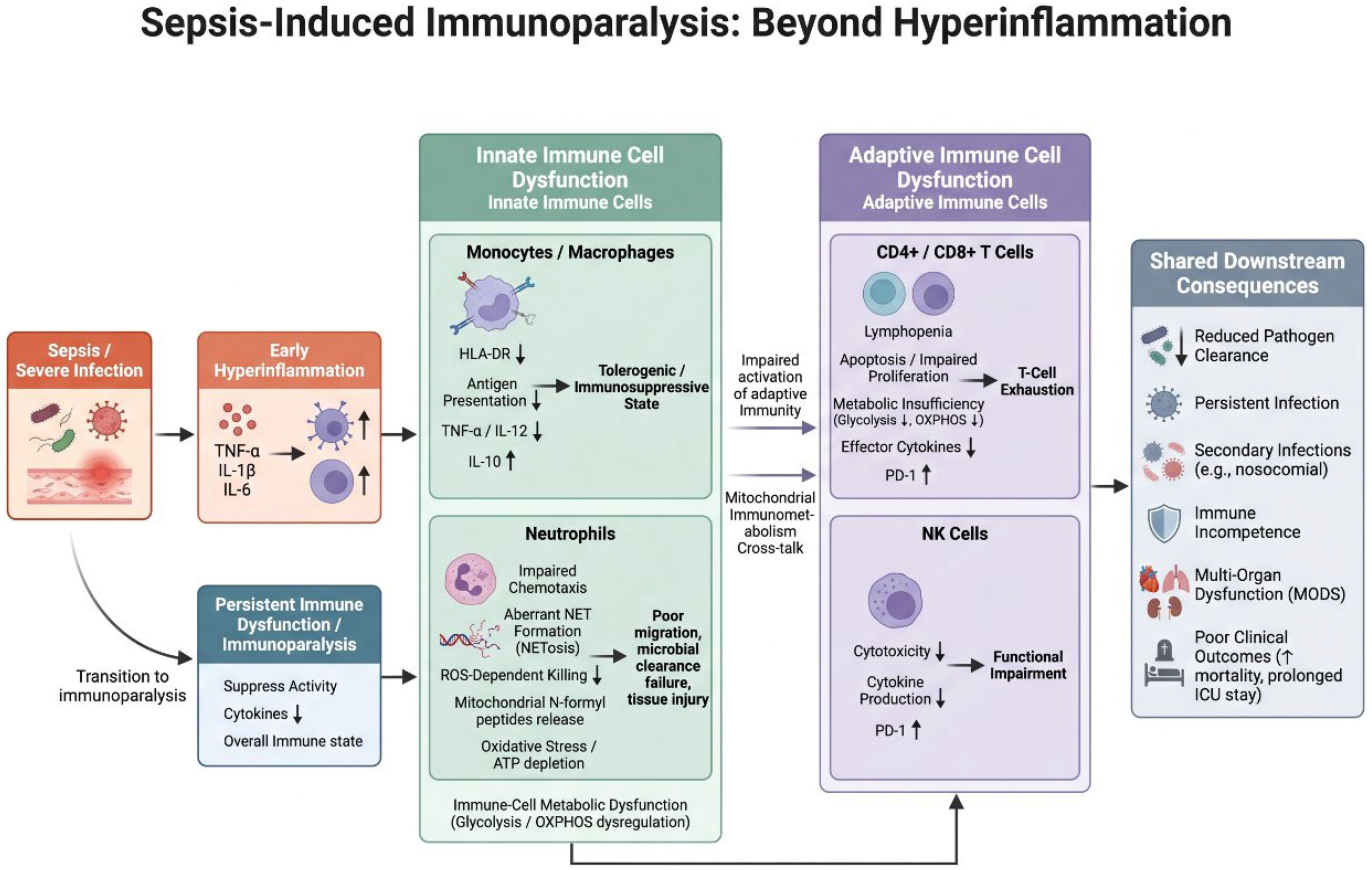
Overview of sepsis-induced immunoparalysis beyond hyperinflammation. Sepsis evolves from early hyperinflammation to immunoparalysis, characterized by monocyte/macrophage deactivation, neutrophil dysfunction, T-cell exhaustion, and NK-cell impairment. These innate and adaptive defects reduce pathogen clearance and increase susceptibility to persistent infection, secondary infections, multi-organ dysfunction, and poor outcomes.

Natural killer (NK) cells, critical for early defense against viral and intracellular pathogens, also display functional impairment in sepsis. NK cells exhibit decreased cytotoxicity and cytokine production, accompanied by upregulation of PD-1, contributing to an immunosuppressive milieu (69, 70). The combined dysfunction of T and NK cells underlies a failure of the adaptive immune system to control persistent infections, and supports the notion that sepsis-induced immunoparalysis is an active, regulated process rather than a passive consequence of overwhelming inflammation. These innate and adaptive immune defects raise an important mechanistic question: how do immune cells become functionally impaired despite persistent infectious stimulation? Mitochondrial immunometabolism provides one explanation for this transition, as discussed in the following sections (71–75). Together, these innate and adaptive immune alterations create a state of profound immunodeficiency that is both measurable and potentially reversible, forming the biological basis for biomarker-guided therapeutic strategies. **Figure 1** provides an overview of the immune transition from early hyperinflammation to sepsis-induced immunoparalysis. It highlights how innate and adaptive immune-cell dysfunction, including monocyte/macrophage deactivation, neutrophil impairment, T-cell exhaustion, and NK-cell dysfunction, converges to impair pathogen clearance and worsen clinical outcomes.

## 3 Mitochondria as central regulators of immune-cell fate

### 3.1 Mitochondrial control of immune-cell metabolism, signaling, and fate

Mitochondria are no longer regarded merely as bioenergetic organelles responsible for ATP generation (76–79). In immune cells, mitochondria function as central signaling hubs that integrate metabolic cues, pathogen-derived signals, redox stress, inflammatory activation, and cell-fate decisions. The metabolic state of immune cells is tightly linked to their functional phenotype. Resting and memory immune cells often rely on oxidative phosphorylation and fatty acid oxidation to maintain long-term survival and immune surveillance, whereas activated immune cells frequently increase glycolysis to support rapid proliferation, cytokine production, and effector responses. Mitochondria therefore help determine whether immune cells remain quiescent, become activated, differentiate into effector subsets, acquire regulatory properties, or undergo apoptosis.

ATP production through oxidative phosphorylation is one of the most fundamental mitochondrial functions (80–84). Adequate ATP supply is required for antigen processing, phagocytosis, cytokine secretion, cell migration, cytotoxic granule release, and lymphocyte clonal expansion. In monocytes and macrophages, mitochondrial metabolism influences antigen presentation and the balance between inflammatory and tolerogenic phenotypes. In T cells, mitochondrial respiration supports survival, memory formation, and sustained effector activity. In natural killer cells and neutrophils, mitochondrial function contributes to cytotoxicity, chemotaxis, and antimicrobial responses. Thus, disruption of mitochondrial energy metabolism can directly impair immune-cell competence and promote features of immunoparalysis.

Mitochondria are also major sources of reactive oxygen species. At physiological levels, mitochondrial ROS act as signaling molecules that regulate innate immune activation, inflammasome signaling, cytokine production, and T-cell receptor signaling. However, excessive mitochondrial ROS can damage proteins, lipids, and nucleic acids, leading to oxidative injury, mitochondrial membrane depolarization, and cell death. In sepsis, this balance is frequently disrupted. Persistent oxidative stress may impair immune-cell responsiveness and contribute to both inflammatory tissue damage and immune exhaustion. Therefore, mitochondrial ROS have a dual role: they are required for antimicrobial signaling, but when uncontrolled, they can drive immune dysfunction. Another important mitochondrial function is the regulation of mitochondrial damage-associated molecular patterns. Mitochondrial DNA is structurally similar to bacterial DNA because of its evolutionary origin and contains unmethylated CpG motifs that can activate innate immune sensors when released into the cytosol or extracellular space (85–88). During severe infection or cellular stress, mitochondrial injury may promote the release of mtDNA, cardiolipin, N-formyl peptides, and other mitochondrial components. These molecules can amplify inflammation, activate pattern-recognition receptors, and alter neutrophil and monocyte responses. Kwon and colleagues showed that circulating mitochondrial N-formyl peptides contribute to sepsis-associated neutrophil dysfunction, suggesting that mitochondrial-derived signals may directly connect tissue injury, immune dysregulation, and impaired pathogen clearance (60).

Mitochondrial quality control is equally essential for immune-cell homeostasis. Mitochondrial dynamics, including fission and fusion, allow cells to adapt to changing metabolic demands and remove damaged mitochondrial segments. Excessive fission may generate fragmented and dysfunctional mitochondria, whereas impaired fusion can limit mitochondrial repair and bioenergetic efficiency. Mitophagy, a selective form of autophagy that removes damaged mitochondria, prevents the accumulation of dysfunctional organelles and limits excessive ROS generation and mtDNA release. The PINK1/Parkin pathway is one of the best-characterized mechanisms of mitophagy. When mitochondria lose membrane potential, PINK1 accumulates on the outer mitochondrial membrane and recruits Parkin, initiating the clearance of damaged mitochondria. In immune cells, efficient mitophagy is necessary to preserve mitochondrial fitness, restrain inflammasome activation, and maintain balanced immune responses.

### 3.2 Shared mitochondrial abnormalities in sepsis-induced immune dysfunction

During sepsis, mitochondrial homeostasis is disrupted by pathogen-associated molecular patterns, inflammatory cytokines, hypoxia, oxidative stress, metabolic stress, and microvascular dysfunction. These insults impair oxidative phosphorylation, decrease ATP generation, increase mitochondrial ROS production, reduce mitochondrial membrane potential, and disturb mitochondrial dynamics and mitophagy. As a result, immune cells may enter a state of metabolic insufficiency in which they are unable to mount effective antimicrobial responses despite persistent inflammatory stimulation. This metabolic failure provides a mechanistic basis for the coexistence of inflammation and immunosuppression in sepsis.

Impaired oxidative phosphorylation is a central feature of mitochondrial dysfunction in sepsis (89–92). Reduced activity of electron transport chain complexes limits ATP production and promotes electron leakage, further increasing mitochondrial ROS. In immune cells, this energetic defect can weaken phagocytosis, antigen presentation, cytokine production, and lymphocyte proliferation. Importantly, immune-cell dysfunction in sepsis is not simply caused by cell loss; surviving cells may remain numerically present but metabolically incompetent. This distinction is particularly relevant to immunoparalysis, where immune cells often display reduced functional responses despite ongoing infection or systemic inflammation.

Mitochondrial ROS accumulation further amplifies immune dysregulation. Excessive ROS can damage mitochondrial membranes and respiratory-chain proteins, leading to a vicious cycle of worsening mitochondrial injury (93–97). ROS also promote the release of mtDNA and other mitochondrial danger signals, which may activate innate immune pathways and inflammasomes. Chen and colleagues identified a mitophagy-related landscape in sepsis and highlighted PHB1 as a regulator of NLRP3 inflammasome activation, supporting the concept that defective mitochondrial quality control can drive inflammatory signaling (98). This finding is important because inflammasome activation may contribute not only to early hyperinflammation but also to later immune exhaustion and tissue injury.

Mitophagy failure has emerged as a particularly important mechanism linking mitochondrial damage to sepsis-associated immune dysfunction (99–103). Li and colleagues showed that macrophage migration inhibitory factor suppresses mitophagy by disturbing the PINK1/Parkin interaction in sepsis-associated acute kidney injury. This suggests that inflammatory mediators can directly interfere with mitochondrial quality control, allowing damaged mitochondria to accumulate and sustain pathological signaling. Similarly, Chen and colleagues reported that hydrogen sulfide may regulate macrophage phenotypic changes through PINK1/Parkin-mediated mitophagy in sepsis-related cardiorenal syndrome (104). These studies indicate that mitophagy does not merely remove damaged mitochondria; it actively shapes macrophage phenotype, inflammatory output, and organ-specific immune responses during sepsis.

Recent work has further linked macrophage signaling pathways to mitochondrial quality-control defects. Zheng and colleagues demonstrated that macrophage Notch1 drives septic cardiac dysfunction by impairing mitophagy and promoting NLRP3 activation (105). This finding connects a classical immune-cell signaling pathway with mitochondrial dysfunction and inflammasome activation, reinforcing the view that mitochondrial damage is integrated into broader immune regulatory networks. In this framework, defective mitophagy permits accumulation of dysfunctional mitochondria, excessive ROS generation, and inflammasome activation, ultimately contributing to immune-cell dysfunction and organ injury.

Mitochondrial membrane potential is another important indicator of mitochondrial integrity. Loss of membrane potential reflects impaired proton-gradient maintenance and is often associated with defective ATP production, increased susceptibility to apoptosis, and activation of mitophagy pathways. In lymphocytes, reduced mitochondrial membrane potential may contribute to apoptosis and impaired clonal expansion. In monocytes and macrophages, mitochondrial depolarization may limit antigen presentation and alter cytokine responses. In neutrophils, mitochondrial damage can affect chemotaxis, oxidative responses, and cell-death programs. Thus, mitochondrial depolarization provides both a mechanistic and potentially measurable feature of immune-cell dysfunction in sepsis. Together, these shared mitochondrial abnormalities provide the mechanistic basis for the cell-specific immune defects discussed below. Rather than affecting all immune cells uniformly, mitochondrial dysfunction produces distinct consequences depending on the metabolic requirements and immune functions of each cell subset.

It is important to interpret these mitochondrial abnormalities according to their level of evidence. In experimental models, interventions that alter mitophagy, mtROS, or mitochondrial respiration can support a causal role for mitochondrial dysfunction in immune-cell impairment. In human sepsis, however, many mitochondrial readouts, such as circulating mtDNA, OCR, mitochondrial membrane potential, and mtROS, are primarily associative or biomarker-level findings. These markers may reflect immune-cell dysfunction, tissue injury, shock severity, or systemic metabolic stress rather than proving direct causality. Therefore, mitochondrial dysfunction should be viewed as a context-dependent contributor and amplifier of sepsis-induced immunoparalysis, while its causal role requires validation through longitudinal sampling, cell-specific assays, and interventional studies.

### 3.3 Immunometabolic reprogramming beyond ATP depletion

Beyond ATP depletion and glycolytic compensation, sepsis-associated metabolic reprogramming involves remodeling of the tricarboxylic acid cycle and differential dysfunction of electron transport chain complexes (106, 107). In activated monocytes and macrophages, succinate accumulation can act as an immunoregulatory metabolite that stabilizes HIF-1α and promotes IL-1β-associated inflammatory signaling (108, 109). By contrast, itaconate, generated through immune-responsive gene 1, inhibits succinate dehydrogenase/complex II and restrains excessive inflammatory activation through effects on succinate oxidation, oxidative stress, Nrf2 activation, and inflammasome regulation (110–112). These metabolites therefore provide a mechanistic link between mitochondrial metabolism and immune phenotype rather than serving merely as passive markers of bioenergetic stress.

Different electron transport chain complexes may also shape immune-cell fate in distinct ways. Complex I dysfunction limits NADH oxidation, alters the NAD+/NADH balance, and promotes mitochondrial ROS production, thereby impairing neutrophil antimicrobial activity and lymphocyte survival (61, 113). Complex II, which also functions as succinate dehydrogenase in the TCA cycle, connects succinate metabolism to mitochondrial respiration and inflammatory signaling (114, 115). Complex III is an important source of mitochondrial ROS involved in antimicrobial and inflammatory signaling, whereas complex IV dysfunction reflects impaired oxygen utilization and may reinforce HIF-1α-dependent glycolytic adaptation under hypoxic septic conditions (113, 116–119). These metabolic signals converge on HIF-1α, AMPK, and mTOR pathways: HIF-1α promotes glycolytic and inflammatory programs, AMPK senses energetic stress and supports mitochondrial adaptation, and mTOR coordinates anabolic metabolism required for immune-cell activation (120–122). The balance among these pathways differs across immune-cell subsets and helps explain why sepsis can produce both hyperinflammation and immunoparalysis.

### 3.4 Temporal dynamics of mitochondrial dysfunction: from hyperinflammation to immunoparalysis

Mitochondrial dysfunction contributes to sepsis-induced immune dysregulation in a time-dependent manner. During the early hyperinflammatory phase, mitochondrial ROS, mtDNA release, N-formyl peptides, succinate accumulation, and defective mitophagy can amplify innate immune activation by promoting pattern-recognition receptor signaling, HIF-1α stabilization, and NLRP3 inflammasome activation (98, 114, 123). In this context, mitochondrial stress initially functions as a danger-amplifying signal that supports antimicrobial defense but also increases tissue injury and cytokine production. However, when mitochondrial damage persists, the same pathways may shift from inflammatory amplification to immune exhaustion and functional paralysis. Sustained mtROS production damages respiratory-chain proteins and mitochondrial membranes, leading to impaired oxidative phosphorylation, ATP depletion, loss of mitochondrial membrane potential, and reduced metabolic flexibility (68, 124). Persistent mtDNA or mitochondrial peptide release may chronically stimulate innate immune pathways, but chronic stimulation can promote endotoxin tolerance, impaired antigen presentation, reduced cytokine responsiveness after ex vivo stimulation, and compensatory anti-inflammatory signaling (125–127). Thus, mitochondrial danger signals may be pro-inflammatory in the early phase but immunosuppressive when exposure becomes prolonged or unresolved.

**Figure 2:**
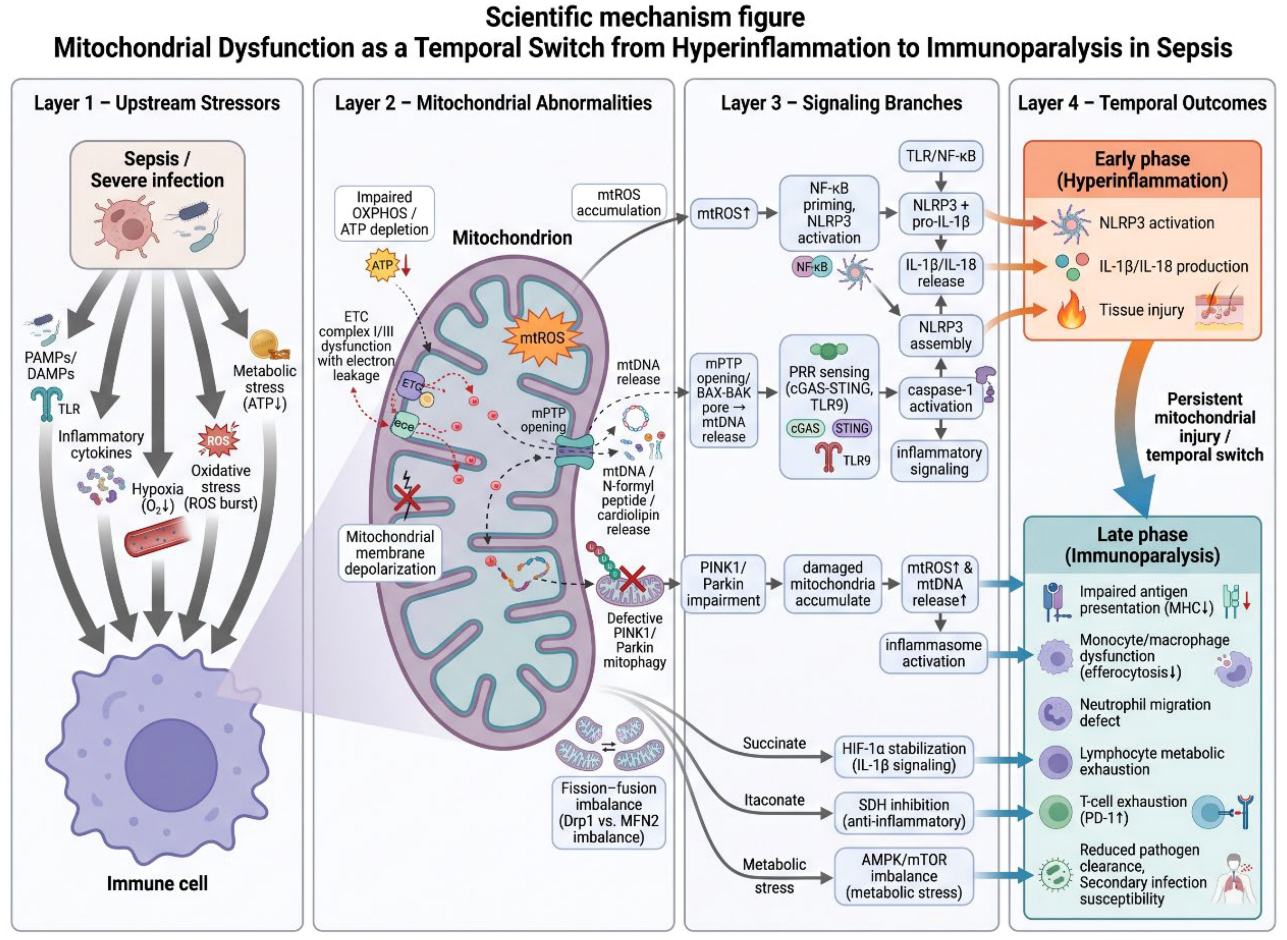
Mitochondrial dysfunction as a temporal switch in sepsis-induced immune dysfunction. Sepsis-associated stress disrupts OXPHOS, ETC activity, membrane potential, mitophagy, and mitochondrial DAMP containment. Early mtROS, mtDNA release, succinate–HIF-1α signaling, and NLRP3 activation amplify inflammation, whereas persistent mitochondrial injury promotes bioenergetic failure, AMPK/mTOR imbalance, impaired antigen presentation, lymphocyte dysfunction, and immunoparalysis.

Several mitochondrial alterations may therefore act as molecular switches in the transition from hyperinflammation to immunoparalysis. First, transient mtROS supports inflammatory signaling, whereas persistent mtROS causes oxidative injury, mitochondrial depolarization, and immune-cell dysfunction. Second, adaptive mitophagy initially limits mitochondrial damage, whereas defective or exhausted mitophagy permits accumulation of damaged mitochondria and sustains pathological inflammasome activation (98, 128, 129). Third, mitochondrial DAMP release may acutely activate host defense, but chronic DAMP exposure can promote immune tolerance, lymphocyte apoptosis, and impaired pathogen clearance (123, 125). Fourth, metabolic signaling may shift from HIF-1α- and mTOR-driven inflammatory activation toward AMPK-dominated energy-stress responses, reduced anabolic capacity, and impaired effector-cell function. These temporal switches help explain how mechanisms such as Notch1-driven NLRP3 activation, MIF-mediated mitophagy suppression, and mtROS-driven inflammatory signaling can contribute not only to early hyperinflammation but also to later immunoparalysis (98, 129). **Figure 2** summarizes how sepsis-associated mitochondrial injury links early inflammatory amplification to later immune paralysis. The figure highlights key mitochondrial switches, including mtROS accumulation, mtDNA release, defective mitophagy, succinate–HIF-1α signaling, NLRP3 activation, and AMPK/mTOR imbalance.

## 4 Mitochondrial dysfunction in innate immune cells during sepsis

### 4.1 Monocytes and macrophages

Monocytes and macrophages are central regulators of innate immunity and play a crucial role in coordinating the host response to infection (130–132). In these cells, the mitochondrial quality-control defects described above mainly translate into impaired antigen presentation, altered cytokine output, and inflammasome dysregulation. For example, Li et al. (2024) demonstrated that macrophage migration inhibitory factor disrupts PINK1/Parkin-dependent mitophagy, resulting in damaged mitochondrial accumulation, excessive mtROS production, and enhanced NLRP3 inflammasome activation. Similarly, Zheng et al. (2025) showed that macrophage Notch1 signaling aggravates septic cardiac dysfunction by impairing mitophagy and promoting NLRP3 activation (105). Metabolic reprogramming further determines whether monocytes and macrophages adopt inflammatory, tolerant, or immunosuppressive phenotypes. Succinate accumulation favors HIF-1α-dependent IL-1β production and supports an inflammatory macrophage program, whereas itaconate limits succinate oxidation through inhibition of succinate dehydrogenase/complex II and may restrain excessive inflammasome activation. During prolonged sepsis, disruption of this succinate–itaconate balance may contribute to the coexistence of inflammatory signaling and impaired antigen presentation, linking metabolic remodeling to macrophage deactivation and immunoparalysis. These findings indicate that defective mitochondrial quality control does not merely reflect cellular stress, but actively reshapes macrophage inflammatory and immunosuppressive phenotypes during sepsis.

### 4.2 Neutrophils

Neutrophils are the first line of defense against bacterial invasion and are critical for pathogen clearance, but their functions are highly energy-dependent and sensitive to mitochondrial perturbations (133–137). In sepsis, neutrophils exhibit mitochondrial reactive oxygen species (mtROS) accumulation and defects in mitochondrial complex I activity, which impair oxidative phosphorylation and energy production. Kwon et al. (2024) demonstrated that excessive circulating mitochondrial N-formyl peptides in sepsis impair neutrophil chemotaxis and ROS-dependent microbial killing, linking mitochondrial-derived damage signals to functional neutrophil exhaustion (60).

Experimental evidence suggests that complex I-related mitochondrial dysfunction and mtROS accumulation may promote regulated cell-death pathways, including PANoptosis, in sepsis-related settings. However, whether this mechanism occurs broadly in circulating human neutrophils during sepsis remains to be clarified. This cell-death program not only reduces the number of functional neutrophils but also releases pro-inflammatory intracellular contents, further exacerbating tissue injury and immune dysregulation (61, 138). Dysfunctional neutrophils therefore contribute both to inadequate pathogen clearance and to systemic inflammatory pathology, illustrating how mitochondrial injury directly drives innate immune failure in sepsis. Because neutrophils rely heavily on glycolysis for ATP generation, mitochondrial dysfunction in these cells should not be interpreted simply as global energy failure. Instead, complex I defects and mtROS accumulation mainly disturb chemotaxis, ROS-dependent microbial killing, NET formation, and regulated cell death.

**Figure 3:**
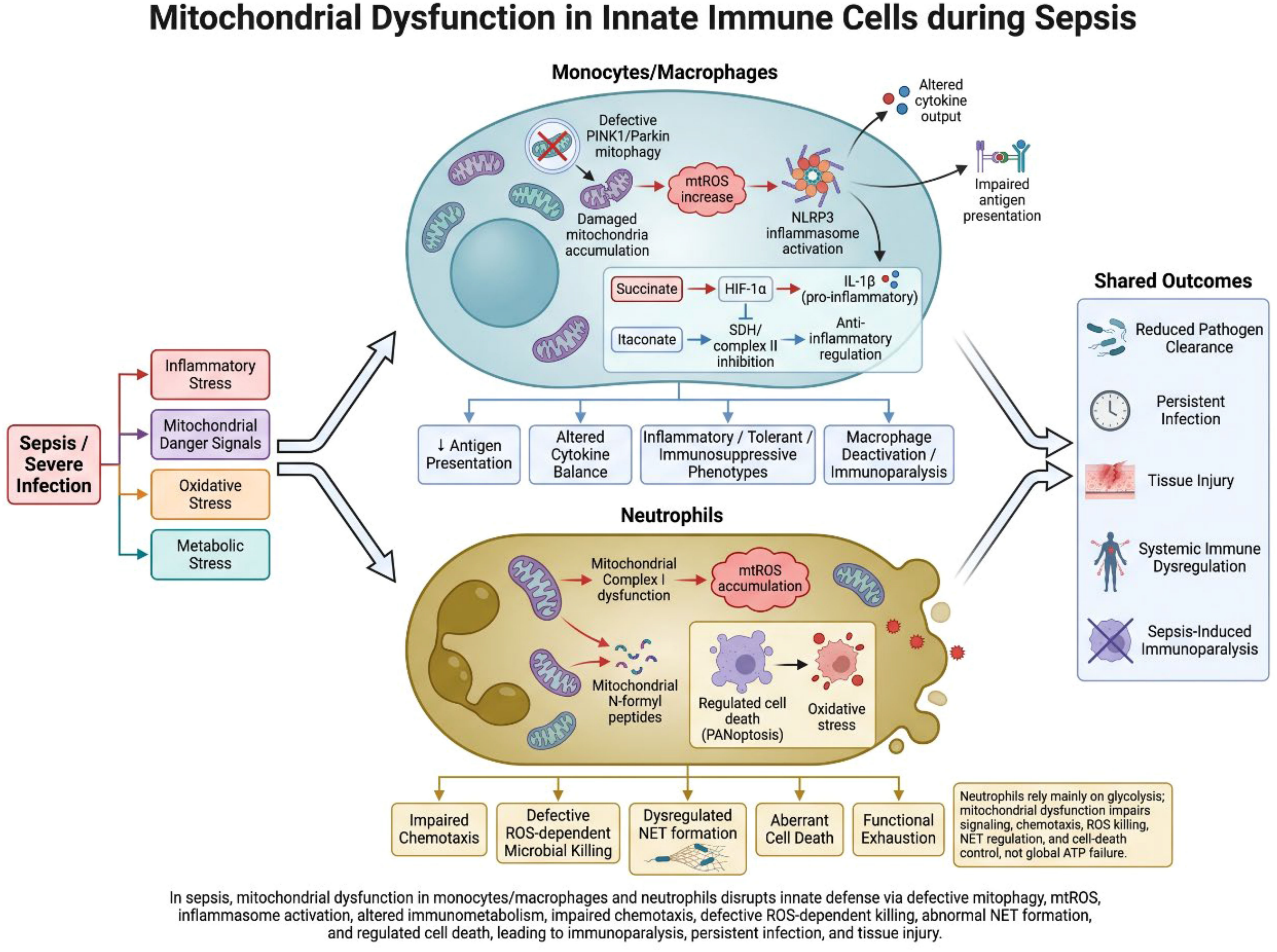
Mitochondrial dysfunction in innate immune cells during sepsis. Sepsis-associated stress disrupts mitochondrial quality control and immunometabolism in monocytes/macrophages and neutrophils. Defective mitophagy, mtROS accumulation, inflammasome activation, altered succinate–itaconate signaling, complex I dysfunction, mitochondrial N-formyl peptides, and PANoptosis impair innate immune defense, promoting reduced pathogen clearance, tissue injury, and immunoparalysis.

Thus, in neutrophils, mitochondrial dysfunction is most relevant to impaired chemotaxis, abnormal ROS-dependent microbial killing, dysregulated NET formation, and aberrant cell death. These neutrophil-specific defects help explain why septic patients may develop persistent infection and tissue injury even when circulating neutrophil numbers are preserved. **Figure 3** summarizes how defective mitophagy, mtROS accumulation, inflammasome activation, altered succinate–itaconate signaling, and neutrophil mitochondrial stress converge to impair pathogen clearance and promote immunoparalysis.

## 5 Mitochondrial dysfunction in adaptive immune cells

### 5.1 T cells

Adaptive immunity is profoundly affected during sepsis, with T lymphocytes exhibiting both quantitative and qualitative defects that contribute to immunoparalysis. CD4+ T cells undergo extensive metabolic reprogramming in response to sepsis-associated stress (139–145). Normally, T-cell activation requires a coordinated switch between oxidative phosphorylation (OXPHOS) and glycolysis to meet energy and biosynthetic demands. In septic patients and experimental sepsis models, reduced mitochondrial fitness has been associated with altered lymphocyte bioenergetics, including reduced OXPHOS capacity and compensatory glycolytic adaptation. However, when these findings are derived from PBMCs or mixed lymphocyte populations, they should not be interpreted as definitive T-cell-intrinsic mitochondrial defects. While glycolysis can transiently support effector functions, persistent mitochondrial impairment limits ATP availability, reduces NAD+/NADH ratios, and generates excessive ROS, collectively resulting in impaired cytokine production, diminished proliferative capacity, and functional suppression (146, 147).

In T cells, the consequences of mitochondrial metabolic stress are shaped by AMPK–mTOR signaling. AMPK is activated by energetic stress and can support mitochondrial homeostasis, fatty acid oxidation, and stress adaptation, whereas mTOR promotes anabolic metabolism, glycolysis, and effector T-cell activation (148). During sepsis, persistent mitochondrial injury may uncouple these adaptive pathways: inadequate OXPHOS and ATP production activate stress responses, while sustained inflammatory and checkpoint signals impair mTOR-dependent proliferation and effector differentiation (149, 150). HIF-1α may further promote glycolytic adaptation, but this shift is insufficient to maintain durable T-cell expansion and cytokine production when mitochondrial fitness is impaired (151, 152). Thus, T-cell exhaustion in sepsis reflects not only reduced energy availability but also dysregulated metabolic signaling through AMPK, mTOR, and HIF-1α.

CD8+ T cells are similarly compromised. Mitochondrial injury promotes T-cell exhaustion, characterized by upregulation of inhibitory receptors including PD-1 and PD-L1, impaired cytotoxic granule release, and reduced interferon-γ production. These exhausted T cells fail to efficiently clear pathogens, contributing to sustained infection and secondary immunodeficiency (68). Mechanistically, the interplay between mitochondrial energy deficits, ROS accumulation, and metabolic signaling pathways such as mTOR and AMPK may contribute to CD4+ and CD8+ T-cell dysfunction, although direct subset-specific causality remains incompletely established in human sepsis. Collectively, these metabolic and mitochondrial alterations explain why adaptive immune responses are markedly impaired despite ongoing antigenic stimulation.

### 5.2 NK cells & B cells

Natural killer (NK) cells, critical for early cytotoxic responses against virally infected and transformed cells, also display functional impairment during sepsis (153–156). Mitochondrial dysfunction in NK cells leads to decreased cytotoxicity and impaired cytokine secretion, a phenotype further exacerbated by upregulation of PD-1, which reinforces immunosuppressive signaling and limits NK-cell effector functions (62, 70). The combination of energy depletion, elevated ROS, and inhibitory receptor signaling creates a metabolic bottleneck, rendering NK cells incapable of effective pathogen clearance.

B cells, particularly PD-L1-positive plasma cells, play an unexpected role in sustaining adaptive immunosuppression (157–159). In both septic patients and experimental models, PD-L1-expressing plasma cells interact with T lymphocytes, inhibiting proliferation and effector cytokine production. This mechanism represents a crosstalk between humoral and cellular immunity, whereby adaptive immune suppression is actively reinforced by metabolic and checkpoint-mediated signals (63, 70). PD-L1-positive plasma cells have been shown to suppress T-lymphocyte responses in patients with sepsis and in mouse sepsis models, supporting an active role of humoral immune populations in adaptive immunosuppression. However, direct evidence linking B-cell or plasma-cell mitochondrial dysfunction to PD-L1 induction in sepsis remains limited. Therefore, the relationship among plasma-cell metabolism, mitochondrial stress, and PD-L1-mediated suppression should currently be regarded as a mechanistic hypothesis requiring cell-specific validation.

These adaptive immune defects indicate that mitochondrial injury does not produce a uniform suppressive phenotype. Instead, it constrains each lymphocyte subset according to its functional demands: proliferation and cytokine production in T cells, cytotoxicity in NK cells, and checkpoint-mediated suppression in PD-L1-positive plasma cells.

### 5.3 Regulatory T cells and myeloid-derived suppressor cells

Regulatory T cells (Tregs) are an important but previously underemphasized component of sepsis-induced immunosuppression. During sepsis, expansion or functional reinforcement of Tregs can suppress effector T-cell activation through IL-10, TGF-β, CTLA-4, and metabolic competition (160). From a mitochondrial perspective, Tregs differ from conventional effector T cells because they are more dependent on oxidative phosphorylation, fatty acid oxidation, mitochondrial membrane integrity, and redox balance to maintain survival and suppressive function (161–163). Therefore, mitochondrial remodeling may not simply impair all T-cell responses uniformly. Instead, mitochondrial stress may weaken effector T-cell proliferation while preserving or enhancing regulatory programs, thereby shifting the adaptive immune balance toward immunosuppression (164).

**Figure 4:**
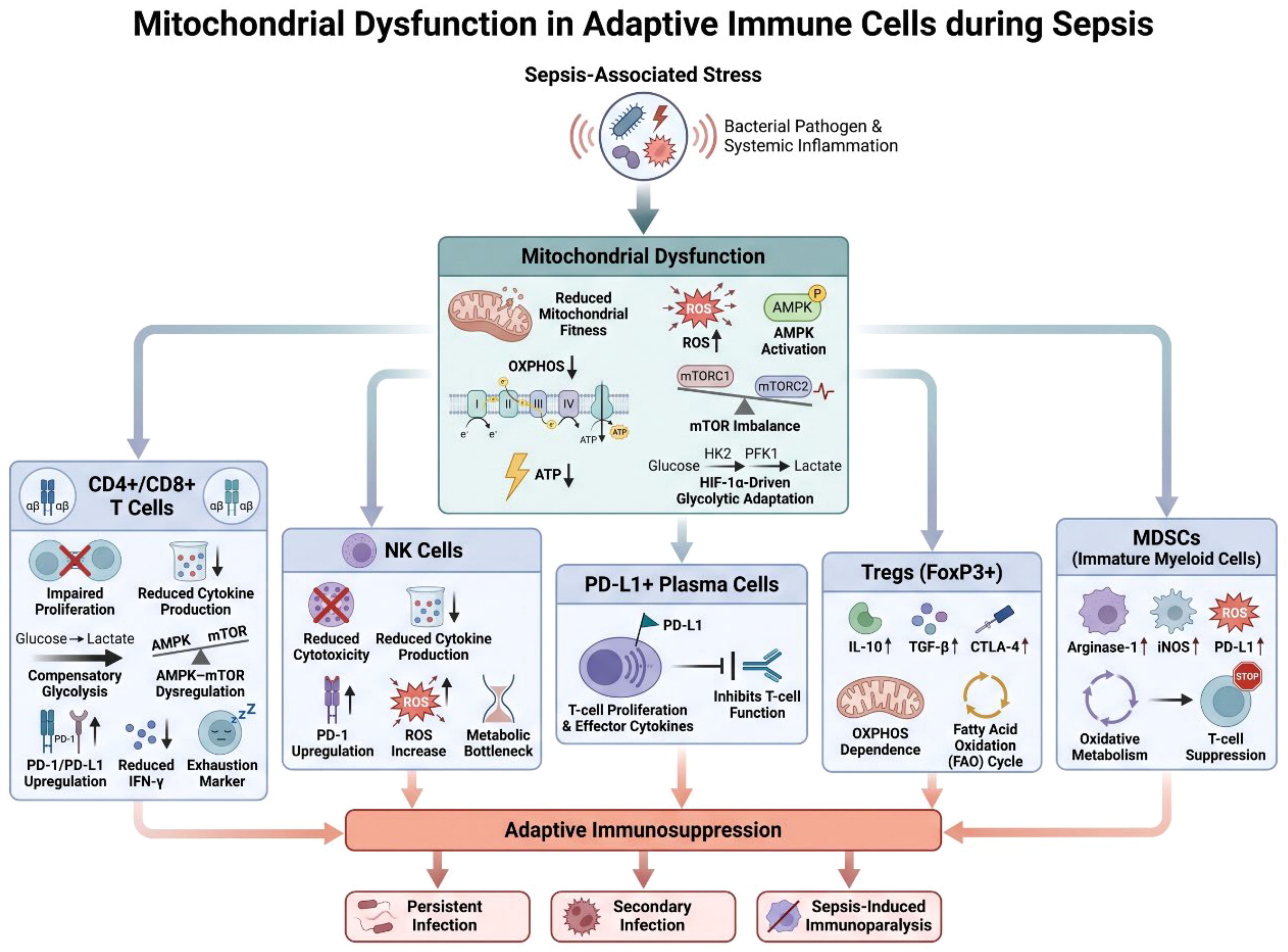
Mitochondrial dysfunction in adaptive immune cells during sepsis. Sepsis-associated stress disrupts mitochondrial fitness and metabolic signaling in lymphoid and suppressive immune subsets. AMPK–mTOR imbalance, HIF-1α adaptation, ROS accumulation, and checkpoint signaling impair T-cell and NK-cell effector functions, while Tregs, MDSCs, and PD-L1-positive plasma cells reinforce adaptive immunosuppression, promoting persistent infection and immunoparalysis.

Myeloid-derived suppressor cells (MDSCs) also represent a central cellular mechanism of sepsis-induced immunoparalysis. MDSCs expand during sepsis and have been associated with clinical worsening, secondary infections, and mortality (165, 166). Their suppressive activity involves arginase-1, inducible nitric oxide synthase, reactive oxygen species, PD-L1 expression, and metabolic competition with T cells (167). Mitochondrial remodeling may further support MDSC survival and suppressive activity by regulating oxidative metabolism, mitochondrial ROS production, and resistance to apoptosis. Recent evidence also suggests that reduced oxidative metabolism and mitochondrial content in sepsis-associated MDSCs may be linked to adverse clinical outcomes, indicating that MDSC mitochondrial fitness may serve as both a mechanistic node and a potential biomarker. Including Tregs and MDSCs therefore broadens the adaptive immune framework from effector-cell exhaustion alone to active immune suppression mediated by regulatory lymphoid and myeloid populations. **Figure 4** summarizes how mitochondrial dysfunction reshapes adaptive immunity in a subset-specific manner during sepsis. The figure highlights the contrasting consequences of mitochondrial remodeling, with impaired effector function in T cells and NK cells but preserved or reinforced suppressive activity in Tregs, MDSCs, and PD-L1-positive plasma cells. **Table 1** summarizes the key mitochondrial alterations observed in innate and adaptive immune cells during sepsis and their functional consequences.

**Table 1:** Mitochondrial dysfunction in immune cells during sepsis: mechanisms, functional consequences, and evidence levels.

Immune cell type	Mitochondrial/metabolic alteration	Functional consequence	Key mechanisms	References (PMID)	Evidence level/translational readiness
Monocytes	Impaired PINK1/Parkin-mediated mitophagy, mitochondrial depolarization, mtROS accumulation	Reduced antigen presentation, decreased mHLA-DR, increased IL-10, impaired ex vivo cytokine response	NLRP3 inflammasome activation, Notch1 signaling, defective mitochondrial quality control	Chen 2023 (PMID: 37359543); Li 2024 (PMID: 38956064); Chen 2024 (PMID: 37971696); Zheng 2025 (PMID: 40414862)	Human observational/ex vivo evidence for monocyte dysfunction; animal and *in vitro* evidence for detailed mitochondrial mechanisms
Macrophages	Accumulation of dysfunctional mitochondria, defective mitophagy, altered mitochondrial dynamics	Skewed polarization, inflammasome overactivation, impaired cytokine balance, tissue inflammatory injury	mtROS accumulation, NLRP3 activation, MIF-mediated mitophagy suppression, Notch1-related mitochondrial injury	Chen 2023 (PMID: 37359543); Li 2024 (PMID: 38956064); Chen 2024 (PMID: 37971696); Zheng 2025 (PMID: 40414862)	Mainly animal and *in vitro* mechanistic evidence; limited direct human macrophage-specific validation
Neutrophils	Complex I deficiency, mtROS accumulation, mitochondrial DAMP exposure	Impaired chemotaxis, abnormal NET formation, defective microbial killing, PANoptosis	Mitochondrial N-formyl peptides, complex I-dependent oxidative stress, mtROS-mediated cell death	Kwon 2024 (PMID: 39107254); Wang 2024 (PMID: 38942784); Chung 2025 (PMID: 41516209)	Human observational/ex vivo evidence for neutrophil dysfunction; animal/*in vitro* evidence for mitochondrial causal mechanisms
CD4^+^ T cells	Reduced OXPHOS, compensatory glycolysis, mtROS accumulation, altered mitochondrial mass	Reduced proliferation, impaired cytokine secretion, increased apoptosis or exhaustion-like dysfunction	AMPK–mTOR imbalance, HIF-1α-dependent glycolytic adaptation, mitochondrial insufficiency	Assis 2024 (PMID: 39015145); Pang 2022 (PMID: 35910903); Stier 2026 (PMID: 41540263)	Human ex vivo and observational evidence; mechanistic interpretation supported by animal/*in vitro* T-cell metabolism studies
CD8^+^ T cells	Reduced mitochondrial fitness, impaired OXPHOS, increased PD-1 expression	Exhaustion, reduced IFN-γ production, impaired cytotoxicity	Metabolic failure, checkpoint signaling, impaired mitochondrial adaptation	Nedel 2023 (PMID: 36795959); Gossez 2025 (PMID: 40155394); Wang 2025 (PMID: 41044025)	Human observational/ex vivo evidence for exhaustion markers; limited direct sepsis-specific mitochondrial mechanistic evidence
NK cells	Mitochondrial depolarization, metabolic stress, increased PD-1 expression	Reduced cytotoxicity and impaired IFN-γ production	Energy stress, mitochondrial dysfunction, checkpoint-associated suppression	Tang 2024 (PMID: 38953031); Gossez 2025 (PMID: 40155394); Lv 2024 (PMID: 38603712)	Human observational evidence for PD-1^+^ NK-cell prognostic value; mechanistic mitochondrial evidence remains limited
B cells/plasma cells	PD-L1 upregulation, mitochondrial stress, altered metabolic state	Suppression of T-cell proliferation and effector cytokine production	PD-L1-mediated T-cell inhibition, metabolic dysregulation	Gossez 2025 (PMID: 40155394); Lv 2024 (PMID: 38603712)	Human and animal evidence for PD-L1^+^ plasma-cell immunosuppression; mitochondrial contribution remains partly hypothesis-generating

Evidence level/translational readiness: Human clinical evidence refers to observational, prognostic, or biomarker studies in septic patients; human ex vivo evidence refers to functional assays using patient-derived immune cells; animal/*in vitro* evidence refers to mechanistic studies in experimental sepsis models or cell systems; hypothesis-generating evidence refers to mechanisms inferred from immune-metabolism literature that still require direct validation in sepsis.

## 6 Clinical and translational biomarkers of mitochondrial immunoparalysis

The identification of reliable biomarkers for sepsis-induced immunoparalysis is critical for early detection, patient stratification, and the development of targeted immunomodulatory therapies. Given the central role of mitochondria in immune-cell metabolism and function, both classical immune markers and mitochondria-specific indicators provide complementary insights into the immunometabolic state of septic patients. Biomarker integration allows for a comprehensive evaluation of immune competence, bridging mechanistic understanding and clinical application. However, the translational value of a biomarker depends not only on its biological relevance but also on its methodological feasibility in critically ill patients. In the ICU setting, an ideal biomarker should be measurable from small-volume peripheral blood samples, require minimal cell isolation, remain stable during short-term ex vivo handling, provide results within clinically actionable time windows, and have reproducible thresholds across laboratories. Therefore, mitochondrial biomarkers should be evaluated together with classical immune markers according to assay complexity, turnaround time, pre-analytical stability, standardization status, and their ability to add clinically useful information beyond established immune phenotyping. Importantly, immune and mitochondrial biomarkers differ substantially in clinical readiness. Markers such as lymphocyte count, lactate, and, in specialized centers, monocyte HLA-DR can be measured relatively rapidly and are closer to real-world ICU implementation. By contrast, OCR, PBMC respirometry, mitochondrial membrane potential, mitochondrial ROS, ATP levels, mitochondrial mass, and single-cell metabolic profiling often require fresh viable cells, rapid processing, specialized platforms, and standardized protocols. Therefore, these assays are currently better considered translational or research-stage tools rather than routine bedside biomarkers. In addition, not all mitochondria-related markers specifically reflect immune-cell mitochondrial dysfunction. Circulating mtDNA and lactate may indicate systemic tissue injury, shock, hypoperfusion, renal dysfunction, or cell death, whereas flow cytometry-based mitochondrial readouts in defined immune-cell subsets may provide more cell-specific information. This distinction is essential when interpreting mitochondrial biomarkers in septic patients.

### 6.1 Clinically accessible immune-function markers

Classical immune-function biomarkers remain foundational for assessing immunoparalysis. Monocyte HLA-DR (mHLA-DR) expression is one of the most widely studied markers, with low levels reflecting impaired antigen presentation and an increased risk of secondary infection (22, 49). Similarly, the expression of PD-1 on T cells serves as an indicator of T-cell exhaustion and functional impairment (69). Additional markers, including CD86, a co-stimulatory molecule essential for T-cell activation, and elevated IL-10, an anti-inflammatory cytokine, provide further insights into the immunosuppressive milieu of sepsis (62, 63). Lymphocyte count, reflecting global adaptive immune competency, is also frequently monitored, as lymphopenia correlates with worse clinical outcomes and heightened susceptibility to secondary infections. Collectively, these immune-function markers offer a practical, readily measurable window into the immune status of septic patients. **Table 2** provides a comprehensive summary of immune-function and mitochondria-related biomarkers that have been investigated in septic patients. The table emphasizes both classical immune markers and mitochondrial/metabolic parameters that can help identify patients at risk of immunoparalysis.

**Table 2:** Immune and mitochondrial biomarkers in sepsis: clinical utility, feasibility, and evidence level.

Biomarker type	Marker	Clinical significance	Measurement	ICU feasibility	Sensitivity/specificity	Standardization status	Evidence level/translational readiness	Major limitations
Immune function	mHLA-DR	Monocyte deactivation, impaired antigen presentation, immune competence	Fresh whole blood flow cytometry	Moderate to high in centers with flow cytometry	Relatively sensitive for monocyte immunoparalysis; moderate specificity	Relatively advanced but still platform- and gating-dependent	Human clinical and ex vivo evidence; relatively close to clinical translation	Requires fresh samples, standardized gating, and defined cut-off values
Immune function	PD-1/PD-L1	T-cell, NK-cell, monocyte, neutrophil, or plasma-cell exhaustion/suppression	Flow cytometry	Moderate	Moderate sensitivity for checkpoint-associated immune suppression; limited specificity	Moderate; cell-subset-specific thresholds remain unclear	Human observational and ex vivo evidence; translationally relevant but not fully standardized	Expression varies by cell type, timing, infection source, and treatment
Immune function	CD86	Co-stimulatory capacity and T-cell activation potential	Flow cytometry	Moderate	Moderate sensitivity; limited specificity as a stand-alone marker	Moderate	Human observational evidence; useful as part of immune phenotyping panel	Influenced by monocyte subset, inflammatory stage, and flow cytometry strategy
Immune function	IL-10	Anti-inflammatory response and compensatory immunosuppression	ELISA or multiplex assay	Moderate to high	Sensitive for anti-inflammatory state; low specificity	Moderate	Human clinical evidence; supportive rather than diagnostic	Elevated in many inflammatory or critical illness states
Immune function	Lymphocyte count	Global adaptive immune suppression and lymphopenia	Complete blood count	High	Sensitive but non-specific	High	Established clinical marker; routine ICU availability	Cannot distinguish T-cell exhaustion, apoptosis, redistribution, or metabolic dysfunction
Immune function	Ex vivo cytokine response	Functional immune responsiveness after stimulation	Whole blood or PBMC stimulation assay	Low to moderate	Functionally informative; potentially more specific for immune tolerance	Low to moderate	Human ex vivo evidence; useful for translational studies	Slow turnaround, protocol variability, and limited routine availability
Mitochondrial	Circulating mtDNA	Mitochondrial injury, DAMP release, systemic stress	Plasma/serum qPCR or ddPCR	Moderate	Sensitive for tissue/mitochondrial injury; low specificity for immunoparalysis	Low to moderate	Human observational biomarker evidence; promising but not disease-specific	Affected by hemolysis, shock, trauma, renal dysfunction, cell death, and sample handling
Mitochondrial	ATP	Bioenergetic status of PBMCs or immune-cell subsets	Bioluminescence assay	Moderate	Sensitive for cellular energy failure; low specificity	Moderate	Human ex vivo and experimental evidence	Influenced by cell number, viability, processing delay, and assay normalization
Mitochondrial	mtROS	Oxidative stress and mitochondrial redox imbalance	Flow cytometry or fluorescent probes	Low to moderate	Sensitive to oxidative stress; limited specificity	Low	Human ex vivo and animal/*in vitro* mechanistic evidence	Probe specificity, dye loading, ex vivo delay, and gating variability
Mitochondrial	OCR/respiratory capacity	Mitochondrial respiration and metabolic reserve	Seahorse extracellular flux assay or PBMC respirometry	Low for routine ICU use	High mechanistic sensitivity; uncertain clinical specificity	Low	Human ex vivo and translational evidence; mainly research-stage	Requires fresh viable cells, cell isolation/enrichment, specialized equipment, and long turnaround time
Mitochondrial	Mitochondrial membrane potential	Organelle integrity and depolarization	JC-1, TMRE, or TMRM flow cytometry	Low to moderate	Mechanistically informative; specificity depends on cell subset	Low to moderate	Human ex vivo and experimental evidence	Requires viable cells; sensitive to dye selection, incubation, temperature, and delayed processing
Mitochondrial	Mitochondrial mass	Mitochondrial content and biogenesis-related status	Flow cytometry or imaging	Moderate in equipped centers	Moderate; reflects content rather than function	Low to moderate	Human ex vivo/translational evidence	Cannot distinguish healthy from damaged mitochondria
Metabolic/multi-omics	Lactate	Glycolytic shift, impaired oxygen utilization, shock severity	Routine biochemical assay	High	Sensitive for severity and hypoperfusion; low specificity for immunoparalysis	High	Established clinical marker; not specific to mitochondrial immune dysfunction	Reflects systemic perfusion/metabolism rather than immune-cell-specific dysfunction
Metabolic/multi-omics	TCA intermediates, including succinate and itaconate	Immunometabolic remodeling, HIF-1α signaling, SDH/complex II activity	Targeted metabolomics	Low for routine ICU use	Mechanistically informative; clinical sensitivity/specificity not established	Low	Mainly translational, animal, and *in vitro* evidence; emerging human relevance	Requires specialized platforms, standardized sampling, and careful interpretation
Metabolic/multi-omics	FAO-related metabolites	Lipid metabolism, T-cell/NK-cell fitness, Treg/MDSC metabolic state	Metabolomics or lipidomics	Low	Mechanistically informative; low current clinical specificity	Low	Primarily translational or hypothesis-generating in sepsis immunoparalysis	Cell-type specificity is difficult to infer from plasma metabolomics

Evidence level/translational readiness: Human clinical evidence refers to observational, prognostic, or biomarker studies in septic patients; human ex vivo evidence refers to functional assays using patient-derived immune cells; animal/*in vitro* evidence refers to mechanistic studies in experimental sepsis models or cell systems; hypothesis-generating evidence refers to mechanisms inferred from immunometabolism literature that still require direct validation in sepsis.

### 6.2 Translational mitochondria-related readouts

Emerging evidence highlights mitochondria-specific biomarkers as sensitive indicators of immune-cell metabolic health. Circulating mitochondrial DNA (mtDNA), released during mitochondrial injury, serves both as a damage-associated molecular pattern and as a marker of systemic mitochondrial stress (60, 168). Functional assays of immune-cell bioenergetics, including ATP content, mitochondrial ROS production, oxygen consumption rate (OCR), and mitochondrial membrane potential, provide quantitative measures of mitochondrial integrity and metabolic competence (68, 169, 170). These parameters have been associated with impaired immune-cell function, but most human data remain observational or ex vivo. Therefore, these biomarkers should be interpreted as indicators of mitochondrial stress or immune-cell metabolic dysfunction rather than definitive proof that mitochondrial dysfunction directly causes immunoparalysis. Importantly, mitochondrial markers may detect immune-cell metabolic deficits even before overt phenotypic exhaustion is observed, offering a potential window for early intervention.

Despite their mechanistic value, mitochondria-related biomarkers face important methodological barriers. OCR and extracellular flux assays provide direct information on immune-cell respiratory capacity, but they usually require fresh viable cells, rapid sample processing, cell isolation or enrichment, specialized equipment, and several hours of assay time. These requirements limit their use as routine ICU tests, especially because immune-cell metabolism can decay rapidly after blood collection or be altered by anticoagulants, temperature, storage time, and cell separation procedures. Mitochondrial membrane potential and mtROS assays are more compatible with flow cytometry, but they also require viable cells and are sensitive to dye selection, incubation conditions, gating strategy, and delayed processing. Circulating mtDNA is technically easier to measure by qPCR or digital PCR and does not require viable-cell isolation, but its specificity for immunoparalysis is limited because mtDNA may also reflect tissue injury, shock, trauma, renal dysfunction, or cell death. Therefore, mitochondrial biomarkers should currently be viewed as complementary tools rather than stand-alone diagnostic markers of sepsis-induced immunoparalysis. Among mitochondria-related biomarkers, cell-specificity varies considerably. Flow cytometry-based measurements of mitochondrial membrane potential, mtROS, mitochondrial mass, or ATP in gated monocytes, neutrophils, T cells, or NK cells can more directly reflect immune-cell mitochondrial status, but these assays are technically demanding and highly sensitive to processing delay, dye conditions, gating strategy, and cell viability. In contrast, plasma mtDNA is easier to quantify but is not specific to immune-cell injury because it may originate from parenchymal tissue injury, endothelial damage, hemolysis, shock, or impaired clearance. Similarly, lactate is clinically useful for assessing shock severity and tissue hypoperfusion, but it should not be interpreted as a specific marker of immune-cell mitochondrial dysfunction. Therefore, mitochondrial biomarkers should be interpreted according to sample source, assay method, and biological specificity.

### 6.3 Metabolic and multi-omics biomarkers for mechanistic stratification

Beyond traditional and mitochondria-specific markers, metabolic profiling provides additional granularity in assessing immune-cell fitness. Elevated lactate levels, reflecting anaerobic glycolysis, often accompany reduced OXPHOS capacity and mitochondrial dysfunction. Alterations in tricarboxylic acid (TCA) cycle intermediates and fatty acid oxidation-related molecules further characterize the bioenergetic status of immune cells and have been linked to functional impairment (138, 170). Advanced techniques, including single-cell metabolomics and PBMC respirometry, allow for high-resolution evaluation of immune-cell metabolic heterogeneity, revealing distinct subpopulations with varying susceptibility to immunoparalysis. Integration of these metabolic and multi-omics datasets with classical immune and mitochondrial markers can generate composite immunometabolic profiles, enabling precise patient stratification and informing personalized immunotherapeutic strategies.

A practical ICU workflow may therefore use a tiered biomarker strategy. First-line immune monitoring could rely on rapidly available and relatively standardized markers, including lymphocyte count, monocyte HLA-DR expression, PD-1/PD-L1 expression, CD86, IL-10, and ex vivo cytokine-response assays when available (171–173). Second-line mitochondrial testing could include circulating mtDNA and selected flow cytometry-based mitochondrial readouts, such as mitochondrial mass, membrane potential, or mtROS, in centers with rapid sample-processing capacity (174, 175). More complex assays, including OCR, PBMC respirometry, and single-cell metabolic profiling, are better suited for translational studies, trial enrichment, and mechanistic phenotyping rather than routine bedside decision-making (176). In this framework, mitochondrial markers would not replace classical immune phenotyping but would help identify whether immune suppression is accompanied by bioenergetic failure, oxidative stress, or defective mitochondrial quality control.

Taken together, the combined assessment of immune function, mitochondrial integrity, and metabolic profiles provides a robust framework for monitoring sepsis-induced immunoparalysis. Such integrated biomarker approaches not only facilitate early detection of immune dysfunction but also help predict clinical outcomes, guide individualized interventions, and evaluate the efficacy of mitochondria-targeted or immunomodulatory therapies.

## 7 Therapeutic implications and translational considerations

Sepsis-induced immunoparalysis represents a major barrier to recovery, as immune dysfunction increases susceptibility to secondary infections and mortality. Given the central role of mitochondrial dysfunction in driving innate and adaptive immune defects, therapeutic strategies aimed at restoring mitochondrial integrity, metabolic competence, and immune-cell function have emerged as promising approaches. These strategies can be broadly divided into mitochondrial-targeted interventions and immunomodulatory therapies. Therapeutic strategies for sepsis-induced immunoparalysis should be interpreted according to their translational maturity. Immune-restorative approaches such as GM-CSF, IL-7, and PD-1/PD-L1 blockade are supported by human immune-phenotyping studies, ex vivo functional data, and early clinical exploration, although they still require careful patient selection. In contrast, mitochondria-targeted approaches, including PGC-1α activation, SIRT3 activation, hydrogen sulfide-related interventions, mitophagy modulation, and mitochondrial transplantation, remain largely preclinical or early translational. For these approaches, unresolved issues include optimal timing, dosing, delivery route, target-cell uptake, durability of immune restoration, safety, and clinically meaningful endpoints. Therefore, mitochondria-targeted therapy should currently be viewed as a mechanistically attractive research direction rather than an established clinical strategy.

### 7.1 Preclinical and translational mitochondria-targeted interventions

Mitochondria-targeted therapies aim to restore energy production, reduce oxidative stress, and improve immune-cell metabolic fitness. Antioxidants, including SIRT3 activators and hydrogen sulfide donors, have been investigated for their capacity to mitigate mitochondrial ROS accumulation and preserve immune-cell function. By enhancing mitochondrial redox homeostasis, these interventions may reduce inflammasome overactivation and limit immune-cell exhaustion.

Pharmacologic PGC-1α activators, such as ZLN005, have also shown potential to improve mitochondrial biogenesis and oxidative metabolism, thereby supporting ATP generation and T-cell and neutrophil effector functions (177). Enhanced PGC-1α activity can promote mitochondrial turnover, sustain oxidative phosphorylation, and reduce excessive ROS production, ultimately stabilizing immune-cell functionality during sepsis. Regulation of mitophagy via the PINK1/Parkin pathway represents another key therapeutic avenue. Studies have demonstrated that defective mitophagy underlies mitochondrial accumulation, ROS elevation, and inflammasome activation in monocytes and macrophages (104, 105, 129). Interventions that restore mitophagic clearance of damaged mitochondria can normalize mitochondrial dynamics, reduce inflammasome-driven cytokine release, and maintain antigen-presentation capacity, thereby enhancing innate immune competence.

Mitochondrial transplantation represents an emerging but still highly experimental strategy for restoring immune-cell bioenergetics in sepsis. Preclinical evidence suggests that delivery of functional mitochondria may improve immune-cell metabolic activity and partially restore phagocytic or cytotoxic functions (138). However, its clinical translation faces several major barriers (178). First, it remains unclear how exogenous mitochondria can be efficiently delivered to circulating monocytes, neutrophils, T cells, or NK cells *in vivo* without rapid uptake and clearance by the reticuloendothelial system, particularly by macrophages in the liver and spleen. Second, the optimal route, dose, timing, source, and quality-control criteria for transplanted mitochondria have not been established (178). Third, extracellular mitochondria may not be immunologically inert. Because mitochondria contain mtDNA, cardiolipin, N-formyl peptides, and other mitochondrial damage-associated molecular patterns, poorly controlled mitochondrial delivery could potentially activate pattern-recognition receptors, amplify systemic inflammation, or worsen immune dysregulation (179, 180). Therefore, although mitochondrial transplantation is conceptually attractive, future studies must define cell-specific delivery methods, biodistribution, persistence, mitochondrial integration, immunogenicity, and inflammatory safety before this approach can be considered for clinical application in septic patients.

### 7.2 Immune-restorative therapies with clinical or *ex vivo* evidence

In parallel with mitochondrial-targeted interventions, classical immunomodulatory therapies aim to correct the functional deficits of both innate and adaptive immune cells (181). Administration of GM-CSF can enhance monocyte HLA-DR expression and restore antigen-presenting capacity, promoting pathogen clearance. IL-7 therapy supports lymphocyte proliferation and survival, counteracting T-cell exhaustion and lymphopenia (22, 49).

Checkpoint inhibition strategies, such as PD-1/PD-L1 blockade, have been investigated to restore T-cell and NK-cell effector functions in sepsis. By relieving inhibitory signaling, these interventions can reverse immune exhaustion, improve cytokine production, and enhance pathogen-specific responses (70). Importantly, these therapies are most effective when applied in a personalized, biomarker-guided manner, as immune phenotyping (e.g., mHLA-DR, PD-1 expression, mitochondrial functional status) allows clinicians to identify patients most likely to benefit and avoid exacerbating hyperinflammatory states.

Taken together, mitochondrial-targeted and immunomodulatory interventions are complementary. While mitochondrial therapies restore cellular energy and redox balance, immunomodulatory agents reinvigorate adaptive and innate immune responses. Integration of these strategies, guided by precise immunometabolic biomarkers, holds promise for mitigating sepsis-induced immunoparalysis and improving patient outcomes. Future clinical studies are warranted to evaluate optimal combinations, dosing regimens, and timing relative to the onset of sepsis and immune dysfunction. **Table 3** lists current and emerging therapeutic strategies aimed at restoring mitochondrial function and immune competence in sepsis.

**Table 3:** Therapeutic strategies targeting mitochondrial dysfunction and immune restoration in sepsis: evidence level, translational readiness, and limitations.

Strategy	Target/mechanism	Effect on immune cells	Evidence level/translational readiness	Clinical feasibility	Major limitations	Key references PMID
Antioxidant/redox-modulating therapy	SIRT3 activation, H_2_S donors, mitochondria-targeted antioxidants	Reduce excessive mtROS, preserve mitochondrial membrane potential, limit inflammasome overactivation, and support immune-cell metabolic fitness	Mainly animal and *in vitro* mechanistic evidence; limited human immune-cell validation	Low to moderate	Optimal timing, dose, target population, and cell-specific effects remain unclear; excessive ROS suppression may impair antimicrobial signaling	37359543; 37971696; 38956064
Mitochondrial biogenesis enhancement	PGC-1α activation, ZLN005-related pathways	Enhance mitochondrial biogenesis, improve oxidative metabolism, and support phagocyte or lymphocyte function	Mainly preclinical animal/*in vitro* evidence; not established in septic patients	Low	Lack of human trials; uncertain timing because mitochondrial biogenesis may be beneficial during recovery but not necessarily during early hyperinflammation	36820088
Mitophagy regulation	PINK1/Parkin pathway, mitochondrial quality-control restoration	Clear damaged mitochondria, reduce mtROS, limit mtDNA release, and normalize inflammasome/cytokine signaling	Animal and *in vitro* mechanistic evidence; limited direct human validation	Low	Mitophagy may have phase-dependent effects; excessive or insufficient mitophagy may be harmful; cell-specific targeting remains unresolved	37359543; 38956064; 40414862
Mitochondrial transplantation	Delivery of functional mitochondria to immune cells	May restore immune-cell bioenergetics and partially improve phagocytic or cytotoxic function	Early preclinical/hypothesis-generating evidence	Very low	*In vivo* delivery to circulating immune cells is unresolved; rapid reticuloendothelial clearance, biodistribution, mitochondrial persistence, immunogenicity, and mitochondrial DAMP-related inflammation are major concerns	41516209; 32887310; 20203610; 29780380
GM-CSF therapy	Myeloid immune restoration; enhancement of monocyte HLA-DR	Increase mHLA-DR expression, improve antigen presentation, and restore monocyte responsiveness	Human clinical and ex vivo evidence; relatively advanced among immune-restorative strategies	Moderate	Requires immune-phenotype-guided patient selection; may be inappropriate in hyperinflammatory phenotypes	36384100; 36384087
IL-7 therapy	Lymphocyte survival and proliferation	Enhance T-cell survival, reverse lymphopenia, and support adaptive immune recovery	Human early-phase/translational evidence; still not routine clinical care	Low to moderate	Optimal patient selection, dose, timing, and safety require further validation	36384087
Immune checkpoint blockade	PD-1/PD-L1 inhibition	Reverse T-cell or NK-cell exhaustion and restore cytokine production/cytotoxicity	Human observational/ex vivo evidence and limited early clinical exploration; not routine	Low	Risk of excessive immune activation; requires checkpoint-positive immunosuppressed phenotype; safety and timing remain uncertain	40155394; 41044025
Personalized immunotherapy	Immune phenotype-guided treatment using ferritin, mHLA-DR, lymphocyte count, PD-1/PD-L1, and mitochondrial readouts	Match immune-stimulatory or anti-inflammatory therapy to patient immune endotype	Human clinical phenotyping evidence; translational framework under development	Moderate in specialized centers	Requires rapid immune monitoring, standardized thresholds, and prospective validation of treatment algorithms	36384100; 36384087

Evidence level/translational readiness: Human clinical evidence refers to randomized trials, prospective clinical studies, or clinical immune-phenotyping studies in septic patients; human ex vivo evidence refers to functional assays using patient-derived immune cells; animal/*in vitro* evidence refers to mechanistic studies in experimental sepsis models or cell systems; hypothesis-generating evidence refers to therapeutic concepts that remain at an early preclinical stage.

## 8 Future perspectives

Despite substantial progress in understanding sepsis-induced immune dysfunction, the clinical translation of mitochondrial immunometabolism remains at an early stage. A major challenge is that immunoparalysis is not a fixed or uniform condition. Instead, immune status changes dynamically during the course of sepsis, and patients may shift between hyperinflammatory, mixed, and immunosuppressive states. Therefore, future research should move beyond single-time-point immune assessment and establish longitudinal monitoring strategies that capture mitochondrial function and immune competence over time. Dynamic measurement of immune-cell mitochondrial activity, including oxygen consumption rate, mitochondrial membrane potential, mitochondrial ROS production, mitochondrial mass, and circulating mitochondrial damage-associated molecular patterns, may provide a more precise view of the immunometabolic trajectory of septic patients. The diagnostic value of peripheral T-cell mitochondrial mass reported by Pang and colleagues supports the feasibility of incorporating mitochondria-related parameters into early sepsis assessment (147).

A second priority is to integrate mitochondrial biomarkers with established immune phenotyping tools. Classical markers such as monocyte HLA-DR, lymphocyte count, PD-1/PD-L1 expression, cytokine profiles, and ex vivo immune-stimulation assays remain clinically valuable, but they may not fully capture the metabolic fitness of immune cells. Combining these markers with mitochondrial readouts could help distinguish patients with true immunoparalysis from those with persistent hyperinflammation or mixed immune states. The PROVIDE trial demonstrated the clinical relevance of immune phenotype-based stratification in sepsis, particularly using ferritin and monocyte HLA-DR to identify macrophage-activation-like syndrome and immunoparalysis. Future studies should build on this framework by adding mitochondrial and metabolic indicators to refine patient classification and guide individualized therapy.

Single-cell and multi-omics technologies offer additional opportunities to define mitochondrial immunoparalysis with higher resolution. Bulk measurements of PBMCs or whole blood may obscure important differences among monocytes, macrophages, neutrophils, T cells, B cells, and natural killer cells. Single-cell transcriptomics, single-cell metabolomics, mass cytometry, flow cytometry-based mitochondrial assays, and spatial immune profiling could identify specific immune-cell subsets with mitochondrial injury, metabolic exhaustion, or checkpoint-mediated suppression. Such approaches may reveal whether mitochondrial dysfunction occurs uniformly across immune compartments or preferentially affects particular cell types. This information is essential for selecting therapeutic strategies, such as mitochondrial-targeted interventions, immune checkpoint blockade, cytokine-based immune restoration, or combined immunometabolic therapy.

Another important future direction is to clarify the causal relationship between mitochondrial dysfunction and immune failure. Current evidence suggests that mitochondrial damage-associated molecules can directly impair immune-cell function. For example, Kwon and colleagues showed that circulating mitochondrial N-formyl peptides contribute to neutrophil dysfunction and that their removal can restore neutrophil activity in experimental sepsis (60). Similarly, emerging work on mitochondrial transplantation suggests that directly restoring mitochondrial function may improve immune-cell metabolism. Chung and colleagues reported that mitochondrial transplantation can restore immune-cell metabolic activity in sepsis models, supporting the therapeutic potential of organelle-level intervention (138). However, additional studies are required to determine the optimal timing, target cell populations, delivery methods, safety profile, and long-term immunological consequences of such strategies.

Early recognition of immunoparalysis is likely to be critical for improving outcomes. Once secondary infections, profound lymphocyte exhaustion, or irreversible organ injury have developed, immune restoration may be less effective. Therefore, future clinical trials should prioritize early immunometabolic risk stratification and define intervention windows in which mitochondrial or immune-directed therapies are most likely to succeed. Importantly, treatment should be guided by immune status rather than disease label alone. Patients with hyperinflammatory features may require anti-inflammatory or organ-protective strategies, whereas those with immunoparalysis may benefit from immune-stimulatory therapy, metabolic support, or mitochondrial repair. Misclassification could lead to ineffective or even harmful interventions.

Finally, the field needs standardized assays and clinically applicable thresholds for mitochondrial immune monitoring. Although measurements such as oxygen consumption rate, mitochondrial membrane potential, mitochondrial ROS, mtDNA, and mitochondrial mass are informative in research settings, their implementation in routine clinical practice remains challenging. Future studies should establish reproducible protocols, validate biomarkers in multicenter cohorts, and determine whether mitochondrial parameters improve prognostic accuracy beyond existing clinical scores and immune markers. Ideally, a practical biomarker panel combining immune phenotype, mitochondrial function, and metabolic status could be developed to identify patients at high risk of persistent immunoparalysis, secondary infection, and mortality.

In summary, mitochondrial dysfunction represents a promising but still underdeveloped dimension of sepsis-induced immunoparalysis. Integrating mitochondrial monitoring with immune phenotyping, single-cell technologies, and clinical risk models may enable more accurate patient stratification and more rational immunometabolic therapy. Early identification and timely correction of mitochondrial immune failure could reduce secondary infections, prevent prolonged immune suppression, and ultimately improve survival in patients with sepsis.

## 9 Conclusion

Sepsis-induced immunoparalysis is a dynamic state of immune failure in which immune cells become functionally impaired despite persistent infectious stimulation. Mitochondrial dysfunction provides a unifying mechanism linking metabolic insufficiency, redox imbalance, danger-signal release, and defective quality control to impaired innate and adaptive immune responses. Importantly, these mitochondrial abnormalities do not affect all immune-cell subsets uniformly, but instead generate distinct functional defects, including reduced antigen presentation in monocytes/macrophages, impaired migration and antimicrobial activity in neutrophils, exhaustion-like dysfunction in T cells, weakened NK-cell cytotoxicity, and checkpoint-mediated suppression by PD-L1-positive plasma cells. Integrating mitochondrial readouts with established immune biomarkers may improve the identification of patients with persistent immunoparalysis and support biomarker-guided immunometabolic therapy. Future studies should validate practical biomarker panels, define clinically applicable thresholds, and determine whether mitochondria-targeted or immune-restorative therapies can improve outcomes in defined septic patient subgroups.
